# *Healthy Vinton*: A Health Impact Assessment Focused on Water and Sanitation in a Small Rural Town on the U.S.-Mexico Border

**DOI:** 10.3390/ijerph120403864

**Published:** 2015-04-07

**Authors:** William L. Hargrove, Patricia M. Juárez-Carillo, Marcelo Korc

**Affiliations:** 1 Center for Environmental Resource Management, The University of Texas at El Paso, El Paso, TX 79932, USA; 2 Pan American Health Organization, WHO, Washington, DC 20037, USA; E-Mail: korcmarc@paho.org

**Keywords:** water quality, sanitation, arsenic, total dissolved solids, *E. coli*

## Abstract

We conducted a Health Impact Assessment (HIA) focused on water and sanitation in Vinton, TX, a small rural town on the U.S./Mexico Border. We present the Vinton HIA as a case study to inform the practice of HIA in rural limited resource communities with higher than average levels of unemployment and poverty, and limited infrastructure. Household surveys, focus groups, and interviews provided quantitative and qualitative data on water sources and quality, sanitation practices, and community health. We found that some of the current water sources in Vinton did not meet drinking water standards for total dissolved solids and arsenic; the majority of septic tanks were not managed properly; and there was a short-term risk of water scarcity due to prolonged drought in the region. Prevalent ailments reported by participants included stomach problems, diarrhea, and skin problems. These ailments can be related to arsenic and/or biological organisms in water. The positive direct and indirect health impacts of improved water and sanitation in Vinton included: reduced gastrointestinal illnesses and skin disorders; improved water quality, quantity, and pressure; reduced risks from failing septic systems; increased property value; potential economic growth; and enhanced quality of life. The negative direct and indirect impacts included: residents’ initial and monthly costs; increased property taxes; increased debt by local government; and the need for ongoing support from changing elected decision makers. The unique challenges in completing this HIA included: (a) limited available data; (b) a culture of fear and distrust among residents; (c) residents’ lack of education, awareness, and civic discourse regarding water and sanitation issues and their impact on public health; and (d) lack of civic discourse and participation in the democratic process. An important outcome of the HIA was the characterization of local water supplies, which motivated and empowered the community members to become more involved in civic discourse concerning their water supplies. Results are transferable to similar low-income rural communities worldwide where residents are lacking in information about their water supplies and in political “voice”.

## 1. Introduction

Since the first Health Impact Assessment (HIA) was conducted in the U.S. in 1999 [1], over 225 HIAs have been completed in 36 states [2]. HIA has become an important strategy for assessing direct and indirect public health impacts of infrastructure improvement and policy change decisions, not only in the U.S. but also internationally [3]. A recent review commissioned by the U.S. Environmental Protection Agency (USEPA) grouped U.S. HIAs into four sectors of interest including: Transportation, Housing/Buildings/Infrastructure, Land Use, and Waste Management/Site Revitalization [4]. No published HIAs in the U.S. have yet focused on water and sanitation, a common infrastructure need in resource-poor communities on the U.S./Mexico border. Of particular interest are inequities associated with inadequate water access, water quality, and sanitation in the border region. Many rural communities in this region resemble those of developing countries in many respects, especially those designated as *colonias* [5]. *Colonias* are defined by the state of Texas as unincorporated residential areas along the Texas-Mexico border that lack basic infrastructure, such as potable water and sewer systems, electricity, paved roads, and safe and sanitary housing. Little is known about actual physical environmental factors that may be associated with adverse health outcomes in border *colonias* of Texas. The bi-national US-Mexico Environmental Program: Border 2012 [6] and Healthy Border 2010 [7] programs have identified access to drinking water and sanitation services as one of the most significant physical environmental determinants of health in rural areas along the US–Mexico border. VanDerslice [8] highlighted a lack of information and analysis regarding the quality of these services in the most underserved areas of the region.

The relationship of water access, water quality, and sanitation with public health is well documented in the developing world [9,10,11]. More recently, Neumann *et al.* [12] surveyed households in Can Tho City, Vietnam and found that incidence of illnesses were highly correlated with available sanitation, source and availability of drinking water, and perceived water quality. Unfortunately, water quality was not measured in their study, but results from their household surveys clearly demonstrated that for an urban setting like Can Tho City, “improved water supplies” requires not only adequate water and sanitation infrastructure, but also delivery of good quality water. A discussion of water and sanitation needs is not only relevant for water-poor countries such as Vietnam but also for wealthy countries with water-poor communities such as occurs in many rural areas of the U.S. For example, Korc and Ford [13] showed the need for this discussion in Texas along the border with Mexico using the Water Poverty Index. Their results, like those of Neumann *et al*. [12], also highlighted the fact that water infrastructure is not enough. Other important considerations include water quality, technical capacity of water providers, and the capacity of the community itself to manage and respond to water issues and environmental impacts. People living on the U.S. side of the border, many of whom are recent immigrants, need to be engaged and empowered to take responsibility for managing environmental and health conditions at the household level, including access to potable water. 

We conducted a HIA focused on water and sanitation in the Village of Vinton, TX, a small rural town on the U.S./Mexico Border. The goals of our HIA were to: (1) assess water quality of current water supplies and the health of residents; (2) identify the potential health impacts of improved water and sanitation infrastructure; (3) inform the decision of the community and local government to implement the proposed infrastructure projects; and (4) inform the decision of potential donors to provide funding for the projects. Our assessment focused on both direct and indirect determinants of health in relation to the proposed infrastructure projects. Socioeconomic and community development determinants of health included individual household costs associated with the improvements, neighborhood improvement and property values, impact on retail and manufacturing businesses, and perceived quality of life.

Our HIA was unique because it is one of the first in the U.S.: (1) focused on water and sanitation; (2) conducted in a predominantly Hispanic community; (3) located in a small peri-urban community instead of a large urban area; and (4) conducted on the U.S./Mexico border. We present here the Vinton HIA as a case study with practical goals aimed at the stakeholders in the community of Vinton, but we also present the results and lessons learned from the HIA process in this challenging region. The border region presents some unique challenges in implementing HIA, because: (1) it cuts across many jurisdictional boundaries, (2) it is largely bilingual; and (3) many of the small cities and towns are resource poor and limited in capacity [14,15]. We will discuss how we overcame some of these challenges and share lessons learned in conducting this HIA. Thus, with this case study we have the additional goal of informing the practice of HIA in resource poor communities in both the developed and developing world. 

## 2. Experimental Section 

### 2.1. Case Study Description

**Figure 1:**
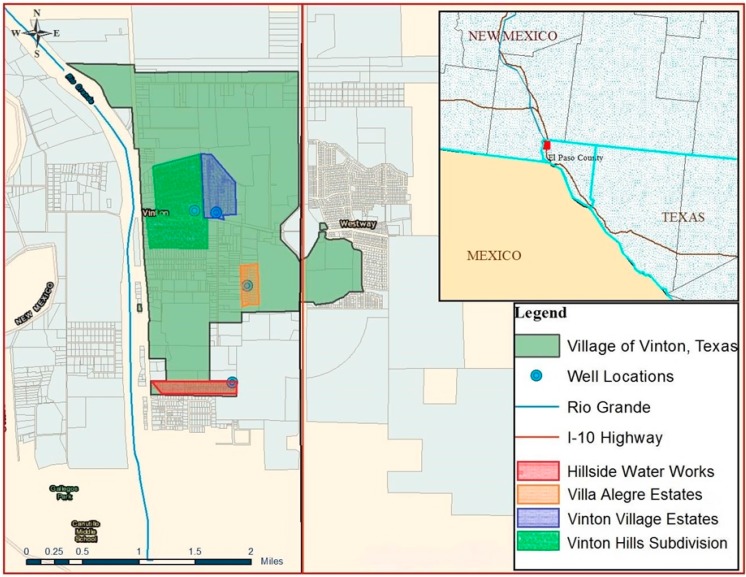
Map showing location and limits of Village of Vinton, and several small privately owned public water supplies.

Vinton is in far west Texas, bounded on the north by the city of Anthony, Texas, on the south by the city of El Paso, on the west by the Rio Grande river, and on the east by Interstate 10 (Figure 1), and has about 2000 residents. It was incorporated as a village in 1962. Since it is incorporated and has an elected village council and mayor, it does not qualify in Texas as a *colonia.* The village lacks a suitable water supply system and depends on septic tanks for sanitation; thus it shares some similar characteristics with a *colonia.* It has a predominantly Hispanic population, a high incidence of unemployment and poverty, and limited infrastructure. The majority of residents rely on privately owned small public water supplies and domestic wells for potable water and other household needs.

Before conducting the HIA, contamination from arsenic (As) and industrial pollutants was suspected with possible drinking water exceedances and elevated risk of exposure for residents. Contamination from failing septic systems and open cesspools was also suspected, contributing to risk of exposure to pathogens and related diseases such as dysentery, hepatitis, typhoid fever, and gastrointestinal illness. Open standing water from failing septic systems and cesspools is also associated with vector-borne diseases such as dengue fever and West Nile virus. No data were found regarding the incidence of these diseases in Vinton. 

#### 2.1.1. Water System.

There are seven major sources of water in Vinton, including: (1) five privately owned small public water supplies from local wells (each of these qualifies as a “public water system” under the definition used by USEPA, which requires at least fifteen service connections); these five small systems collectively serve 63% of the households in Vinton; (2) domestic wells serve about 20% of the households; and (3) El Paso Water Utilities (EPWU), about 17% of the households. Engineering plans were completed in July 2012, describing a new water system that would connect the entire village to the EPWU system. The estimated cost for the project was about $15 million. The City Council and the residents were considering a decision whether or not to implement the project at the time that we conducted the HIA. A number of agencies also were considering technical and financial assistance for the project, including the Border Environment Cooperation Commission (BECC), the North American Development Bank (NADB), Texas Water Development Board, and the U.S. Department of Agriculture Rural Development Agency (USDA/RDA). 

#### 2.1.2. Wastewater Collection System.

The proposed sanitation infrastructure project would provide wastewater collection for the community. An engineering feasibility study was completed in November 2011 and revised in January 2012, which described the project. The plan called for wastewater to be discharged to the EPWU system and conveyed to their Northwest Wastewater Treatment Plant. The estimated cost for the project was about $20 million. In 2012, the City Council voted to delay the project based on high costs, but plans to reconsider the project in 2015. Potential funders of the project also include BECC/NADB, USDA, and others. 

### 2.2. Methodology

Our methodology incorporated the minimum elements and practice standards for health impact assessment as recommended by the North American HIA Practice Standards Working Group [16]. Our HIA followed the six recommended steps of Screening, Scoping, Assessment, Recommendations, Reporting, and Monitoring [2,4,16]. We chose this methodology because it is the prevailing methodology recommended in the U.S. A special challenge of our HIA was that there were limited data available on public health, quality of current water sources, condition of septic systems, or knowledge and perceptions about water and sanitation at the household level in this community. Thus, there was the need to collect such information firsthand through surveys, interviews, focus groups, and public meetings. We conducted the HIA over a period of 12 months, April 2013–March 2014, with most of the new data collection and survey work being conducted over the period of June–August, 2013. We used a community-based participatory approach that drew heavily on the information and perceptions of community members and other key stakeholders. Community members and other stakeholders participated in identifying the key questions and important issues for inquiry in addition to directly informing the assessment. Their input was collected through a variety of means including public meetings, focus groups, and one-on-one discussions. The community participatory approach allowed us to identify key health and environmental issues and indicators, while ensuring a rich contextual understanding and attention to important cultural issues. All protocols were approved by the Institutional Review Board for Human Subjects Research at the University of Texas at El Paso (#409441-2). All subjects gave their informed consent for inclusion before they participated in surveys, interviews, and focus groups. 

**Figure 2:**
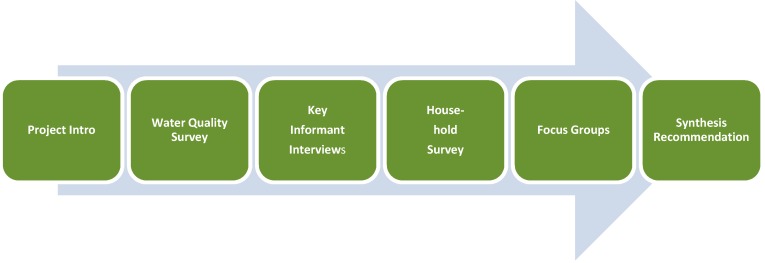
Summary of our assessment process.

Due to space limitations, we are not going to discuss the Screening and Scoping steps in detail but focus instead on the assessment itself, our findings, and the relevance of our work to the practice of HIA in similar circumstances. A flow diagram showing the assessment process is provided in Figure 2. A brief description of each step follows. 

Project Introduction. We introduced our project to the community through a public meeting at the local elementary school and a presentation to the City Council. We hosted a training workshop on HIA methodology for local participants and stakeholders. 

Water Quality Survey. We conducted a water quality survey by collecting a total of 113 tap water samples over the summer of 2013. Samples were collected using USEPA guidelines [17]. Samples were place on ice and delivered to the analytical lab within 24 h of collection. The major water sources, the number of household connections for each, the number of water samples collected from each, and the number of households surveyed from each water source are provided in Table 1. Vinton Mobile Home Park was not sampled because the owner did not agree to let us sample or survey any households in this mobile home park. In Texas, domestic wells are not registered or reported. We estimated the number of domestic wells by subtracting the total number of connections to public supplies from the total number of households. We sampled at least eight households from each of the six major water sources at each time for a total of 96 samples, from 69 households. We also collected 17 samples from local businesses or public places like the public park. EPWU Certified Lab analyzed the samples for total arsenic, chloride, nitrate, sulfate, turbidity, electrical conductivity, total dissolved solids (TDS), temperature, *E. coli*, total coliforms, and pH. Results for chloride, nitrate, sulfate, turbidity, and pH were not abnormal and were within the recommended values for drinking water; thus we do not present these results. But As and TDS were often over the recommended levels; therefore, we focus our results and discussion on As and TDS. Arsenic was determined using EPA Method 200.8 [18] and TDS using EPA Method 2540C [19]. There were a few households that had positive results for either *E.coli* or total coliforms, using EPA’s Standard Methods, SM 9223 A,B [20]. For the few positive detections of *E.coli* or coliform bacteria, we sent additional samples from that household to a certified lab, BioVir Laboratories in Benicia, CA. They used EPA Standard Method 1623 [21] to test for *Giardia* and *Cryptospiridium.* No positive results were obtained for *Giardia* or *Cryptospiridium*. Results for As and TDS were analyzed statistically using one-way ANOVA, and Tukey’s procedure was used to identify significant differences among water sources. 

**Table 1:** Water sources, number of connections, and number of samples.

Water System	Number of Connections	Number of Households with One or Two Water Samples	Number of Surveys	Households with both Water Samples and Survey
Vinton Hills Subdivision	158	17	40	15
Vinton Village Estates	82	9	24	6
Hillside Water Works	52	13	12	10
Vinton Mobile Home Park	36	0 ^**^	0 ^**^	0 ^**^
El Paso Water Utilities	92	12	22	6
Villa Alegre Estates	22	3	5	2
Private Wells	113 ^*^	15	18	10
Total (% of Total Households)	555	69 (12%)	121 (22%)	49 (9%)

Notes: ^*^ Estimated number of households with private wells based on the 555 total households reported by the U.S. Census 2010. ^**^ Owner did not give permission to collect water samples or conduct household surveys.

Key Informant Interviews. We conducted eleven key informant interviews with stakeholders from federal, state, and local government agencies; professionals in the health, education, and religious sectors; business leaders; and community leaders. These individuals had key information and perceptions about public health and the potential impacts of the proposed projects on health by virtue of their professional position. We used the pathway diagrams and research questions from our scoping step (not shown) to develop appropriate questions for these interviews. In turn, these interviews informed the household survey that we developed. 

Household Survey. We completed 121 household surveys, representing 22% of the total households in Vinton. The households were selected utilizing a stratified sampling method combined with systematic sampling of individual households. First, we identified each sampling stratum according to the water source including the four privately owned water supplies, domestic wells, and EPWU. Secondly, all households within each stratum were numbered and 20% of these households were randomly chosen for sampling. If no one came to the door for the selected household, we moved to the house directly to the right. One adult in each household was interviewed. The interviews lasted 29 min on average and 51% of the participants preferred Spanish as the language to conduct the interview. The survey focused on current conditions and practices with respect to water and sanitation, perceptions about the proposed projects, and potential health impacts. It was comprised of a combination of dichotomous, categorical, and Likert scale questions. 

For comparative purposes, we surveyed a “reference” population in Westway, a nearby community with similar demographics but which has had EPWU water for at least 10 years. In Westway, we surveyed 50 households, by dividing the map of the community into five roughly equal portions and using stratified random sampling in each subunit, similar to Vinton. We used the same survey instrument, modified slightly to account for the current water and sanitation conditions (only EPWU for water and sanitation). Results from both surveys were analyzed using chi-square tests to test the association of water source with either perceptions or self-reported illnesses, and to test for differences between communities. 

Focus Groups. After we collected water quality data, key informant interviews and household surveys, we conducted four focus groups to share results, ask further questions, and collect perceptions about the results that had been collected to date. The focus groups were used to validate results and begin to discuss conclusions and recommendations. We conducted focus groups representing each of the following sectors/groups: local professionals, business leaders, agency stakeholders, and community leaders. We invited all individuals in the respective category that we were aware of who were from Vinton or worked in Vinton. 

Synthesis/Recommendations. After the fieldwork was completed, we synthesized our results, identified options for improving water quality and sanitation management and presented them to community members and decision makers, and formulated recommendations, based on feedback from stakeholders. We recognized that our assessment process did not result in definitive cause and effect data. We quantified the prevalence of self-reported diseases and health issues most commonly associated with poor water and sanitation and made extrapolations, based on best available evidence, to the potential health impact of the proposed projects on health outcomes. More definitive associations between poor water and sanitation with public health outcomes demand rigorous epidemiological studies beyond the scope of this assessment. 

## 3. Results and Discussion

### 3.1. Demographic Characteristics of Vinton and the Participants in Household Surveys in Vinton and Westway 

From U.S. Census data [22,23], the population of Vinton is 1,971, 94% of whom are Hispanic (90% of the Hispanics are of Mexican origin), 5% white, and 1% other. Thirty-seven percent of the residents are foreign born (Latin America, mostly Mexico). The median household income is $32,206 (the statewide average in Texas is $48,259). Thirty-seven percent of households live with less than $25,000 per year and 18% of the population is living in poverty. Fifty-four percent of residents have completed high school. The median age is 27 years, which is significantly below the Texas state average of 41 years and over a quarter of the population is under 18 years of age. The total number of housing units is 555, of which 96.6% are occupied (536), and 82.1% are owner-occupied. (440). The average family size is four members per household.

The demographics of the participants in the survey in Vinton and Westway are presented in Table 2. The demographic characteristics of the households surveyed in Westway were similar to Vinton with the exception that Vinton households were somewhat more affluent and more of them preferred to communicate in English instead of Spanish. In Vinton, many residents were fearful of our intentions and thought that we were trying to identify undocumented immigrants or were identifying residents who were for or against the proposed infrastructure projects to report to the local government. On average, only about one third of households that we approached agreed to participate in the survey. 

**Table 2:** Demographics of participants in our surveys in Vinton and Westway.

Characteristics	Vinton		Westway	
	Value	%	Value	%
**POPULATION**				
Total households surveyed	121		50	
Median age (respondent)	48		50	
Total population of participating households	485		170	
Population of participating households by age range				
5 years of age or less	53	11	19	11
6 to 17 years of age	98	20	30	18
18 to 64 years of age	300	62	106	62
65 and more years of age	34	7	15	9
Mean school years of education (respondent)	11		9.6	
**HOUSING**				
Average household size	4.0		3.4	
Average years living in this town	15.6		16.7	
**INCOME**				
Median household income estimation	$25,500		$18,000	
Household income by range				
<$10,000–20,000	44	36	29	59
$21,000–40,000	50	41	11	21
$41,000–60,000	11	9	7	14
$61,000–80,000	8	7	3	5
>$80,000	8	7	0	0
**SURVEY**				
Preferred language by participants				
English	59	49	12	24
Spanish	62	51	38	76
Household participation (% of total households contacted who agreed to participate)		58		57

### 3.2. Water Quality Survey

Of the 113 water samples collected, the constituents that exceeded water quality standards were As, TDS, total coliforms, and *E. coli*. No organic industrial pollutants were found. Results for As and TDS are provided in Table 3. The mean level of As for all water sources was 7.8 µg/L. Moderately higher levels of As were found in water from Hillside Water Works, where the mean value was 10.9 µg/L, and 96% of the samples collected exceeded the drinking water standard of 10 µg/L. The highest concentration of As was found in a domestic well (15.8 µg/L). Thirty-two percent of the total samples exceeded the drinking water standard for As (36/113). 

**Table 3:** Arsenic (As) and total dissolved solid (TDS) concentrations and related parameters for different water sources in Vinton.

SAMPLE INFORMATION		ARSENIC ^†^			TOTAL DISSOLVED SOLIDS ^††^		
Water Sources (total Number connections)	Number of Samples (% of total connections)	Range in As Conc. µg/L	Mean As Conc. ± St. Dev. * µg/L	Number Samples >MCL for As (% > MCL)	Range in TDS Conc. mg/L	Mean TDS Conc. ± St. Dev. * mg/L	Number Samples >Standard for TDS (% > MCL)
Hillside Water Works (52)	23 (44%)	7.4–12.3	10.9 ^a^ ± 0.9	22 (96%)	530–830	791 ^b^ ± 59	0 (0%)
Vinton Village Estates (82)	16 (20%)	8.9–11.1	9.5 ^a,b^ ± 0.6	4 (25%)	642–692	666 ^b^ ± 16	0 (0%)
Domestic Wells (113)	22 (20%)	2.6–15.8	8.3 ^a,b^ ± 5.2	9 (39%)	482–1480	832 ^a,b^ ± 217	3 (14%)
El Paso Water Utilities (92)	22 (24%)	5.8–13.0	6.7 ^b^ ± 1.5	1 (5%)	230–802	507 ^b^ ± 94	0 (0%)
Vinton Hills Subdivision (158)	25 (16%)	4.6–5.6	5.0 ^b^ ± 0.3	0 (0%)	850–946	899 ^a,b^ ± 24	0 (0%)
Villa Alegre Estates (22)	5 (23%)	4.6–5.4	4.9 ^b^ ± 0.3	0 (0%)	988–1020	1002 ^a^ ± 13	2 (40%)
Total (519)	113 (22%)	2.6–15.8	7.8 ± 3.0	36 (32%)	230–1480	759 ± 182	5 (4%)

Notes: ^†^ Maximum concentration limit (MCL) for arsenic in drinking water is 10 µg/L. ^††^ Secondary standard for total dissolved solids in Texas is 1000 mg/L, and at EPA is 500 mg/L. * ^(a,b)^ Means followed by the same letter are not significantly different at the 0.05 level of probability using Tukey’s procedure.

The mean level of TDS for all water sources was 759 mg/L (Table 3). The highest level of TDS was found in Villa Alegre Estates (1020 mg/L). Like As, TDS concentration was highest in a sample from a domestic well (1480 mg/L). The secondary standard for TDS in Texas is 1000 mg/L. In the U.S., there is no drinking water standard for TDS. The secondary standard is set primarily as a goal for protecting pipes from corrosion and other non-human health impacts. There could be indirect health impacts of high salt concentration water. For example, washing daily in water with high salt concentration could dry out the skin, causing irritation and discomfort. 

Ten households resulted in a positive detection for total coliforms (9% of samples) and one household was positive for *E. coli* (1% of samples). No viruses or other pathogenic organisms were found (*Giardia*, *Cryptospiridium*, or other oocysts). 

We delivered the water quality survey results, either by mail or in person, in English and/or Spanish, to each household that was sampled and offered to answer their questions. Most participants were not aware of their water quality and claimed that they had never received analyses of their water before.

### 3.3. Household Survey

#### 3.3.1. Practices and Perceptions Regarding Water and Sanitation

Water. The majority of households surveyed in Vinton (70%) bought bottled water to drink, and used their tap water for all other household purposes. In Westway, fewer households purchased bottled water to drink (44%), and the majority drank their tap water from EPWU (56%). We asked survey participants in Vinton to rate taste and their level of trust in the quality of their drinking water on a scale from 1 to 5 with 1 being the worst taste or least trust and 5 the best taste or greatest trust. Chi-square tests were performed to test the association of water source with the level of trust and taste of tap water. Results were significant for the association of water sources with level of trust (x^2^ = 33.1, *p* = 0.0) and taste (x^2^ = 11.6, *p* = 0.17). Results for both taste and trust were in the order bottled water > EPWU > local wells. Residents of Vinton drank more bottled water because generally the water from local wells tastes worse and they have less trust in the water compared to EPWU or bottled water. 

Sanitation. Vinton relies on septic tanks for sanitation, while Westway is connected to EPWU for sanitation. Our survey of households in Vinton demonstrated a poor level of understanding of septic tanks and how they should be maintained. Several household members were unaware that they had a septic tank. Each survey team inspected the site of the septic tank, if known, to observe any foul odors or standing water. If odors or standing water were detected, they considered the condition as “at risk”, and as “no risk” if no odors or standing water were observed. Forty percent of households were deemed “at risk”. The average age of septic tanks was 20 years. Thirty-one percent of residents knew nothing about their septic tank and 67% claimed that they had never received any information on how to maintain it. Forty-one percent of households never had a septic tank inspection and another 32% did not know whether their septic tank had ever been inspected. Additionally, most residents were unaware of the need for a certificate of compliance or how to obtain one. About half of households had never had their septic tank pumped out or did not know when it might have been pumped out. The other half had had their tanks pumped within the last five years.

Costs of Water and Community Attitudes Regarding the Costs of Infrastructure Improvements. The costs of the infrastructure improvements and the monthly costs of water and sanitation were a concern in a community where so many live under the poverty level, which is one of the few potentially negative impacts of the proposed project. Table 4 presents average household expenditures for water, including expenditures for bottled water. Households spent, on the average, almost $23/mo on bottled water. There is a wide range in total expenditures for water, due to how much bottled water is purchased and how much water is used for landscaping and other outdoor uses. In Vinton, the range is $4–324/mo, with a mean value of $53/mo. Households in Vinton spend much less on piped water from private providers compared to EPWU (about $33/mo *vs.* about $59/mo), but if you add the purchased bottled water, the costs are similar ($55.50 *vs.* $59). In Westway, the reported costs included water and sanitation at an average of $71/mo. EPWU charges for sanitation on a prorated basis of water usage. Assuming that sewage costs are about 30% of the total monthly bill, (a reasonable estimate according to EPWU), the costs of water alone in Westway would be on average about $50/mo, or about $9 less than EPWU customers in Vinton and $5 less than households in Vinton purchasing piped water plus bottled water. It appears that if Vinton would be connected to EPWU and households would drink tap water instead of bottled water, their costs for water could be less than they paid at the time of the survey. At the time of the survey, 85% of households in Vinton purchased at least some bottled water to drink. If connected to EPWU, those households that have domestic wells and pay essentially nothing for water will experience higher monthly costs. These higher costs could negatively impact resources available for other health promoting expenditures or activities, and could even result in having less money available for food (especially fresh fruits and vegetables), or health care (especially preventative health care). At the same time, we found that the preponderance of respondents were willing to pay for improvements in water and sanitation (72%–77%). This finding is significant in that it demonstrates that the economics of improved water and sanitation provided by EPWU in terms of monthly costs is not prohibitive, and residents are willing to pay for the additional costs for improved quality and services. 

**Table 4:** Expenditures for water by individual households in Vinton and Westway (U.S. Dollars, $).

**VINTON**					
**Water Source**	**N**		**Mean**	**Minimum**	**Maximum**
El Paso Water Utilities ^1^	22		$58.88	$13	$300
Local Wells/Private Providers ^2^	77		$32.94	$12	$100
Vinton Hills Subdivision	38		$33.39	$12	$86
Vinton Village Estates	22		$27.21	$12	$62
Villa Alegre Estates	5		$28.40	$17	$40
Hillside Water Works	12		$43.92	$20	$100
All Purchased Piped Water ^3^	99		$38.71	$12	$300
Bottled Water ^4^	103		$22.52	$2	$100
Total ^5^	115		$53.12	$4	$324
**WESTWAY**					
**Water Source**	**N**	**Mean**		**Minimum**	**Maximum**
El Paso Water Utilities ^6^	46	$71.11		$35	$175
Estimated Sewage ^7^	46	$21.33		$10	$52
Estimated Water Only ^8^	46	$49.78		$25	$123
Bottled Water ^9^	41	$18.56		$1	$75
Total ^10^	46	$66.32		$25	$145

Notes: ^1^ Cost of water only; no sewage in Vinton; ^2^ All private providers of piped water; excludes domestic wells and EPWU; followed by breakdown for each private provider system; ^3^ All piped water excluding domestic wells; ^4^ All bottled water purchases, including households with domestic wells; ^5^ Total spent on water, including piped and bottled water for 99 households plus 16 households that have domestic wells; ^6^ Includes water plus sewer in Westway; ^7^ Sewage estimated at 30% of the total cost; ^8^ Water estimated by subtracting estimated sewage from total bill; ^9^ All bottled water purchases; ^10^ Total spent on water; piped plus bottled.

#### 3.3.2. Public Health Condition: Self-Reported Illnesses

Health problems reported by any family member within the past 6 months for both Vinton and Westway and that might be related to poor water quality and/or sanitation include: (1) Numbness, cramping, or tingling in fingers, arms, or legs, 30% of respondents in Vinton (could be related to As); (2) Gastro-intestinal problems, 28% of respondents in Vinton (could be related to poor water quality and/or sanitation); and (3) Skin problems, 26% of respondents in Vinton (could be related to As or to high salt content of water which leads to excessive dryness). Gastro-intestinal ailments and skin problems tended to be more prevalent in Vinton compared to Westway but the difference was not significant. There were no reports of serious illnesses related to water or poor sanitation such as methemoglobinemia, hepatitis, or West Nile virus. The results that we obtained represent self- reported illnesses, not necessarily severe enough to seek medical attention, though some did seek medical attention for these illnesses. 

**Figure 3:**
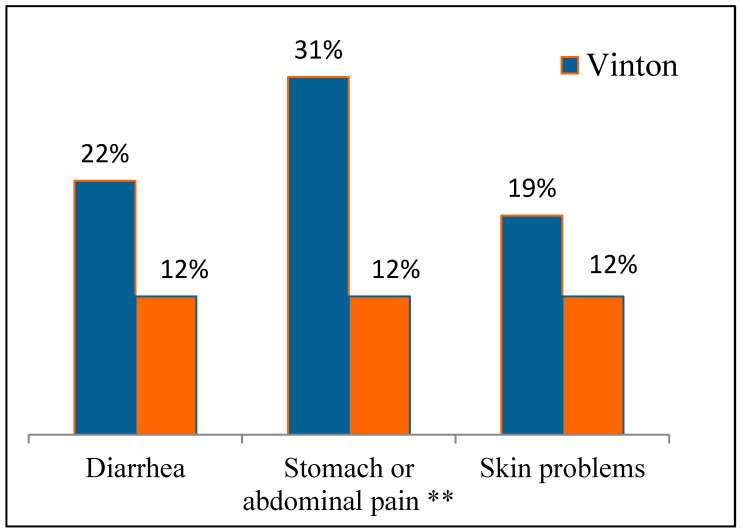
Self-reported ailments in the last 30 days from survey in Vinton and Westway. ** significant difference between communities using chi-square test, *p* = 0.01 (x^2^ = 6.7).

Health problems within the past 30 days are presented in Figure 3. The frequency of gastrointestinal illnesses and skin problems were 22%–31% and 19%, respectively, in Vinton. The prevalence of these ailments tended to be greater in Vinton compared to Westway. In particular, the prevalence of stomach or abdominal pain in Vinton (31%) was significantly greater than in Westway (12%). Since these are self-reported complaints, there are no other reliable data from independent studies with which to compare these frequencies. Seven to 10% of participants reported either children missing school or adults missing work due to gastro-intestinal or skin ailments.

**Figure 4:**
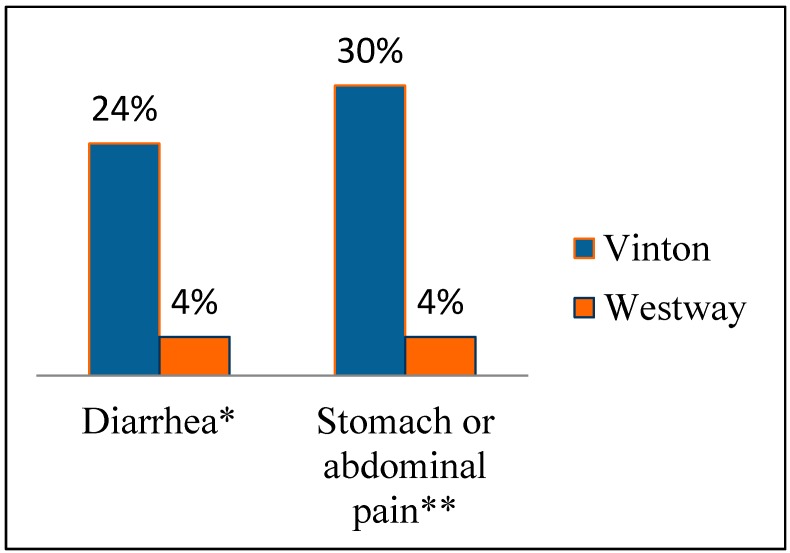
Proportion of households drinking tap water and reporting ailments in the last 30 days. * Significant difference between communities using chi-square test, *p* = 0.03 (x^2^ = 4.91). ** Significant difference between communities using chi-square test, *p* = 0.007 (x^2^ = 7.02).

Figure 4 shows the prevalence of gastrointestinal illnesses and skin problems in the most recent 30 days for households that drink tap water. Similar results were found for ailments reported in the past six months (data not shown). In the case of Westway, all households are served by EPWU, while in Vinton, the results in Figure 4 are for households served by local wells, either domestic wells or small public water systems. These results show that these ailments are much more prevalent in Vinton than in Westway. In particular, the prevalence of gastrointestinal complaints is significantly higher in Vinton for households that drink tap water compared to Westway. Results presented in Figure 3 were for all households whether they drank tap water or not.

**Figure 5:**
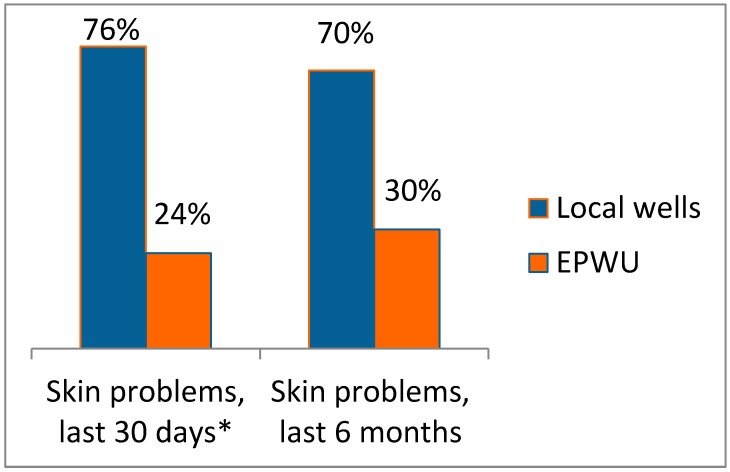
Prevalence of skin problems in relation to water source. * Significant difference between water sources using chi-square test, *p* = 0.04 (x^2^ = 4.5).

Figure 5 shows the prevalence of skin problems in relation to water source, either local wells in Vinton or EPWU in either Vinton or Westway. The prevalence of skin problems where the water source is local wells is about twice that for EPWU as a water source. This difference is statistically significant. The results for gastrointestinal ailments and skin problems when local wells is the water source for drinking and washing point to exposure to arsenic, salts, and coliform bacteria in water as potential determinants of health in Vinton compared to Westway.

### 3.4. Discussion of Contaminants in Water, Health Outcomes, and Relative Risks in Vinton 

#### 3.4.1. Arsenic in Drinking Water

There is a dearth of published studies conducted in the U.S. on health outcomes from As in drinking water; most are from arid and semi-arid areas outside the U.S. According to the World Health Organization (WHO), “acute effects of arsenic exposure include vomiting, abdominal pain, and diarrhea. These are followed by numbness and tingling of the extremities, muscle cramping and death in extreme cases.” [24]. According to research, chronic exposure to arsenic is generally manifested through skin lesions and pigmentation. Other symptoms include peripheral neuropathy, lung and cardiovascular diseases, conjunctivitis, weakness, anemia, diarrhea, hepatomegaly, fetal loss and infant mortality. According to Battacharjee *et al.* [25], 15%–20% of exposed individuals manifest skin lesions. In other studies, the prevalence of unexplained skin rashes was associated with arsenic in drinking water (>10 ppb) [26,27]. In a study conducted in Texas counties along the U.S./Mexico border, Dutton *et al.* [28] found that 3% of individuals living in *colonias* reported skin rashes compared to only 1% of individuals not living in a *colonia*. This could be linked to poor water quality in *colonias.* Recently studies have linked exposure to As in water to respiratory illnesses also, including abnormal lung function among individuals who also have skin lesions [29]. In animal studies, Ramsey *et al.* [30] showed that *in utero* exposure and early life exposure to As in drinking water exacerbated the inflammatory response to influenza A. Most recently, Wasserman *et al.* [31] showed that chronic exposure to drinking water As concentrations as low as 5 ppb negatively impacted the IQ of children and other child development parameters. These published results collectively indicate a potential relationship between arsenic in drinking water and the prevalence of skin rashes, gastrointestinal disorders, and neuropathy in extremities in our assessment. The impact of chronic levels of exposure to As in drinking water on children and pregnant mothers needs more attention, but certainly the recent study by Wasserman *et al.* [31] raises concern about relatively low (5 ppb) levels of exposure to As in drinking water, which is the case for children living in Vinton. Reviewing records of the Texas Commission for Environmental Quality (TCEQ) during the period of 2005–2010, 100% of community wells in Vinton have experienced at least one violation of drinking water standards (most commonly for arsenic). By comparison, in Texas as a whole, 9% of community water supplies reported health-based violations in 2009 [32]. In a national level survey of domestic wells conducted by the USGS, 23% of wells had at least one chemical contaminant above the drinking water standard. 

#### 3.4.2. Total Dissolved Solids in Water

Published studies on health impacts of drinking water exceeding 1000 ppm are very limited. A report from WHO [33] summarizes impacts of total dissolved solids in drinking water on health. In Australia, the risk of ischemic heart disease and acute myocardial infarction were increased in communities with drinking water sources with high levels of soluble salts, calcium, magnesium, sulfate, chloride, fluoride, alkalinity, total hardness, and pH. In the former Soviet Union, cases of inflammation of the gallbladder and gallstones increased with increasing levels of total dissolved solids in groundwater. We hypothesize that high salt content of water used for washing and bathing can result in excessive drying of the skin, skin irritation, and skin rashes and infections, but this has not been proven. More commonly, high TDS levels influence consumers’ acceptability of drinking water because of the taste. Certain TDS components result in excessive scaling in water pipes, heaters, boilers, and appliances.

#### 3.4.3. Bacteria and Other Biological Contaminants

There is ample evidence for negative impacts of bacteria, such as *E. coli* and *Salmonella*, and other organisms, such as *Giardia* and *Cryptosporidium*, in drinking water or even exposure through skin contact with contaminated water on public health. We did not find *Giardia* or *Cryptosporidium* in any of our water samples, but we did find *E.coli* and other coliform bacteria. In the study by Dutton *et al*. [28] in Texas border counties, children living in *colonias* who were 1–5 years in age were much more likely than non-*colonia* children to have had diarrhea in the past two weeks. In *colonias* without water or sanitation, 20% of children <1 year of age had diarrhea in the past two weeks. Again these results could be related to poor water and sanitation in *colonias* compared to communities with access to potable water and sanitation. 

Results from El Paso County in 2013 [34] showed the following prevalence rates related to stomach infections and dysentery:

- *Campylobacteriosis*—716 cases/100,000 people
- *Salmonellosis*—14.5 cases/100,000 people
- *E. coli*—0.24 cases/100,000 people

In a national level survey of domestic wells conducted by the USGS, 34% were positive for total coliform bacteria, and 8% were positive for *E. coli* [8].

We did not collect specific information about the prevalence of parasites in Vinton. However, there are some older data available. In the Canutillo School District where Vinton children attend school, over 22% of participant first graders in elementary schools, Vinton elementary included, were found to be infected with at least one parasite (*i.e.*, *Blastocystis hominis*, *Giardia lamblia*, and *E. coli* among others). Specifically in Vinton, 18.5% of children attending Childress Elementary were infected with a parasite [35]. This prevalence could be associated with poor water quality and/or sanitation. These results are also over 10 years old; the prevalence could be different now.

In a more general study of parasites and other environmentally related infections on both sides of the U.S. Mexico border (El Paso and Ciudad Juarez), of 386 asymptomatic participants, 38.2% had *H. pylori*, 3.3% had *Taenia*, 2.7% had *Giardia*, and 1.9% had *Cryptosporidium* [36]. A study in semi-rural areas of El Paso County reported that 17% of school children tested positive for Hepatitis A [37], but no data were available that were specific to Vinton. Also these studies just report the prevalence of the disorders without any association to a particular environmental exposure.

#### 3.4.4. Relative Risks 

The “relative risk” measures the magnitude of association between a factor (exposure) and the effect (disease) and “indicates the likelihood of developing the disease in the exposed group relative to those who are not exposed” [38]. We calculated the relative risk associated with drinking tap water from local wells in Vinton for various illnesses according to the following equation:

Relative Risk= I_e_ / I_o_ (1)

where, I_e_ = incidence of disease in the exposed group (households drinking water from local wells), defined as: individuals with a self-reported illness/total individuals exposed, and I_o_ = incidence of disease in the non-exposed group (households drinking bottled water or water from EPWU), defined as: individuals with a self-reported illness/total individuals non-exposed. Residents of Vinton who drank tap water from local wells had a relative risk of 8.3 for diarrhea and 11.3 for other gastro-intestinal problems, compared to residents who drank tap water from EPWU (either in Vinton or Westway). 

### 3.5. Other Findings: Results from Focus Groups and Interviews

Through our survey, interviews with key informants, and focus groups, we identified a number of other challenges related to water and sanitation; these are identified and discussed below. 

Manufacturing and Retail Businesses. Community leaders, business leaders, and residents believe that water and sanitation will encourage growth in local retail and manufacturing businesses. From focus groups with representatives from the business sector, we heard that Vinton is considered a “good place to do business”, but without water and sanitation the potential for growth is limited. An increase in retail and manufacturing businesses would also lead to more local jobs for the community. 

Property Values. Improved water and sanitation would likely increase property values. Using a combination of census data, county data, and some survey data, we estimate that property values in Westway, a community similar to Vinton, increased by about 18% over the period from 2000 before they connected to water and sanitation to 2012, a period of 10–12 years after connection. During the same time period, property values in Vinton decreased by about 7%. A negative result of property value increases for owners would be an increase in taxes. We did not attempt to quantify this impact. 

Fire Safety. The main industrial area of Vinton has no fire hydrants. This results in high rates of fire insurance for manufacturing businesses in Vinton and a large concern for fire safety for their employees. The high cost of insurance was a major concern of business leaders in the community. Due to low water pressure, 53% of the fire hydrants in Vinton are non-functional. A number of residential streets also have no fire hydrants. Lack of functioning fire hydrants is a major deterrent to economic and community development.

Water Security. Due to prolonged drought in the region and the reduced flow in the Rio Grande, which is connected to shallow groundwater in the area, residents are fearful that groundwater levels will fall to a level that will make local wells “run dry”. There is a USGS monitoring well in Vinton near the Rio Grande. From their data reported on their website [39], we found that the groundwater level has dropped at least 15 feet in the last year alone. For the period of record (about 25 years), the current water level is in the lowest 10% quartile. 

Recreation Space. Vinton has two public parks that are used sparingly. Neither of the parks has a water fountain or access to drinking water, which could be a factor in limiting their use.

Long-Term Economic and Community Development. Residents and businesses agree that improved water and sanitation will result in long-term public health, economic, and community development benefits. They are willing to pay the cost of connections, but also are concerned about the economic burdens and want to know the exact costs. 

### 3.6. Summary of Existing Conditions, Impacts, Recommendations, and Outcomes

We summarize the existing conditions and their impacts in Table 5. We concluded that current tap water quality in Vinton poses a moderate risk to public health because many of the self-reported ailments can be related to arsenic, salts, and/or fecal contamination of drinking water. Poorly managed septic tanks also pose a moderate risk to public health. During the rainy season, they can produce odors, contaminate the aquifer, and are at risk of overflow. 

Several options were identified to improve the quality of drinking water in Vinton. These are identified and briefly discussed below. 

(1) Treatment of water at the source. Residents could insist that private providers use additional treatment at the supply source to reduce arsenic and salts. This would be expensive. Domestic well owners could chlorinate water stored in their tanks but additional treatment to reduce arsenic and salts at the tank would not be feasible.

(2) Point of use treatment. There are a number of point of use treatment options to reduce salts and arsenic. There are “in-line” filters and other treatment devices based on reverse osmosis that are commercially available at a cost in the range of $200–600/unit. These devices would need to be replaced or recharged once or twice per year. Thus they are also expensive.

(3) Connect to EPWU for water. EPWU draws water from multiple sources, including deeper and more reliable aquifers, maintains adequate water pressure, and complies with drinking water standards. This supplier is more reliable and dependable for the future and increases trust in piped water that can result in less bottled water consumption. 

**Table 5:** Summary of existing conditions and their impacts identified through the assessment.

Existing Condition	Impacts
Poor water quality: As, TDS, Coliform Bacteria	Gastrointestinal ailments Skin problems
Reliance on bottled water for drinking	High cost of drinking water
Poor septic tank management	Risk of overflows leading to: Exposure/gastrointestinal ailments Odors Contamination of groundwater
Inadequate fire hydrants	High cost of fire insurance Inability to fight fires
Lack of local health clinics	Lack of local access to health care, especially preventative care
Lack of water availability at local parks	Lack of drinking water fountains and shade lead to less use
Prolonged drought	Unreliability of water supply; periods of no service
Lack of retail businesses	Lack of economic vitality Poor job opportunities locally

Two options were identified to reduce the health risks associated with poorly functioning septic tanks: (1) an educational campaign on septic tanks and their management, combined with greater regulatory oversight and enforcement; and (2) connection to EPWU for sanitation. 

Discussion of these conditions and options with community members and decision makers resulted in a strong consensus that connection to EPWU was the best long-term solution for the Village of Vinton with respect not only to public health outcomes but also costs, economic development, and overall quality of life in the community.

Based on the assessment findings and the feedback from the community, we summarize the following recommended actions and their predicted outcomes for Vinton:

- Connect to EPWU for water and sanitation; this should lead to improved public health, economic development, and improved quality of life in the community.
- Seek financial assistance for the project from government agencies, including not only assistance for the basic infrastructure but also assistance for individual households to meet the costs of connection; this should lead to reducing the financial burden on the residents, especially those who are disadvantaged by low income and limited resources.
- Install the appropriate number of functioning water hydrants; this should lower the cost of fire insurance and improve public safety.
- Develop a strategic plan for economic and community development; this should improve economic opportunity and overall vitality of the community.
- Develop and implement educational campaigns to educate residents, politicians and decision makers, and youth of the benefits of improving water quality and sanitation in terms of public health; this should improve political and overall community support for the proposed infrastructure projects.
- Develop and implement an educational campaign focused on water conservation measures that might help residents reduce water use, lessening their monthly fees, *i.e.*, improved water efficient appliances, water efficient showerheads and toilets, and water-efficient landscaping; this should lessen the financial burden of higher monthly fees for water for many households.

### 3.7. General Observations and Lessons Learned from this HIA 

Our HIA is unique in that it focused on water and sanitation in a U.S./Mexico border community. Because we conducted this HIA in a rural community that is disadvantaged by low income and limited resources, it has particular relevance to not only other similar communities in the U.S. but also many similar communities in the developing world. We faced a number of unique challenges. We identify below a number of these challenges, how we addressed them, and some additional observations and lessons learned. 

#### 3.7.1. Lack of Data

There is often a lack of readily available data to use in an HIA: (a) in small rural communities compared to larger cities, and (b) for ailments related to poor water quality and sanitation because they are not sufficiently severe to warrant seeking medical care and stem from chronic exposure. The lack of data required us to collect original data and make direct observations for the assessment. This combined with the need to build a level of trust with the community resulted in a much greater time requirement and personnel investment in order to complete this HIA. On the positive side, our HIA provided much needed information on the existing conditions in the community, especially water quality information that was not previously available to community members. This information motivated and empowered the community members to become more engaged in civic discourse related to water and sanitation. 

#### 3.7.2. Culture and Language

In rural border communities, there is a significant number of recent immigrants, both documented and undocumented. Many prefer Spanish to English and because there are many individuals who are not documented, there is a fear and distrust of outsiders asking questions and taking surveys. Some households that we visited feared that we worked for the U.S. government immigration services. Thus they were either not willing to speak with us or maybe not willing to be truthful. In our case, we hired university students who were from the area and who spoke Spanish fluently. Several of the students were from Mexico and Spanish was their first language. This lessened the fear and distrust but did not completely eliminate it. With time, residents became more aware and trusting of our project. 

#### 3.7.3. Building Awareness, Education, Civic Engagement and Discourse

Initially, Vinton residents in general had a poor understanding and awareness of water and sanitation issues and reported feeling marginalized in the local democratic process. Over the years prior to this HIA, the level of trust and communication of residents with their local government had eroded. As a result of the HIA, residents became much more aware of water quality and sanitation issues and their health impacts. The level of civic discourse regarding health, water, sanitation, and the quality of life was heightened. Residents began to attend city council meetings to hear our results and discuss the future of water and sanitation in a public forum. Trust and a modicum of honest and open dialogue began to emerge, initiated by the open communications of the HIA and the sharing of its findings. Public participation through surveys, focus groups, public meetings, fairs, and interviews, became an invaluable opportunity to bring community and other stakeholder perspectives and concerns to the discussion. Finally, the HIA process opened a door for identifying other needs, concerns, and ideas for community improvement. The HIA process catalyzed a “smart growth” project in Vinton and the community began to develop a human security plan.

#### 3.7.4. The Value of HIA to Informed Decision Making 

Through this HIA, the awareness of decision-makers, funders, and other stakeholders regarding the social, political, and economic complexities of water and sanitation systems and their public health outcomes, was also increased. We conducted briefings for key government decision makers at the state and local level, such as county judge, state legislators, and U.S. Congressmen. We also briefed potential funding agencies, such as BECC, USDA-Rural Development Agency, USEPA, the Texas Water Development Board, the Texas Department of Agriculture, and the TCEQ. For all of these decision makers and funders, we provided information on the current situation in Vinton and scientifically sound information on potential impacts of the proposed infrastructure projects. This is seldom done for these kinds of infrastructure projects. It pointed to the value of HIA and how it can inform decisions or validate the benefits of infrastructure projects.

#### 3.7.5. Access to Water, Human Security and Resilience 

Human security is a particularly salient concept for the U.S./Mexico border region. People living on the U.S. side of the border, many of whom are recent immigrants, need to be empowered to take on more of the responsibility for their own health, whether it is preventive behavior, knowing when and how to seek care, or managing health conditions at the household level. Improving human security though self-reliance and self-determination will, in turn, build resilience in the border region. Achieving a “secure border” will not be realized solely though militarization, but will be realized through improving human security and building resilience on both sides of the border. 

#### 3.7.6. University Participation

Local university participation was a positive contribution to the success of this HIA. The HIA provided a very important experiential learning opportunity for university students. The students who participated expressed pride and satisfaction in using their technical training to do something positive in a local community. In addition, the HIA demonstrated not only to Vinton but also to our metropolitan region the power of science-based information in decision-making and the role of the local university in providing unbiased information. 

## 4. Conclusions 

For the community of Vinton, we concluded that improved water and sanitation infrastructure will result in several positive long-term benefits, including: (1) improved public health; (2) reliable water quality, quantity, and pressure; (3) reduced risks from wastewater overflow, odors, and aquifer contamination; (4) economic development; and (5) improved community development and quality of life in Vinton. A negative impact is the cost to households for (1) connection to EPWU for water and the monthly costs for water; (2) connection to EPWU for sanitation, the decommissioning of septic tanks, and the monthly costs for sanitation; and (3) potentially increased property taxes. 

This HIA was unique in several respects, and we had to overcome a number of challenges. These included a lack of readily available data to inform the HIA; a level of fear and distrust in the community stemming from the preponderance of recent immigrants, both documented and undocumented; a lack of education and awareness about water and sanitation and their health impacts in the community; and a lack of civic discourse and reduced participation in the democratic process at the local level. University participation provided a positive impact by engaging local students with appropriate cultural and language skills, while providing them an important experiential service learning opportunity. Nonetheless, our results are relevant and transferable to similar low-income rural communities worldwide where residents are lacking in information about their water supplies and in political “voice”.

The wider implication of this HIA is that it provides an example of how information leads to empowerment and how it operationalizes the concept of access to water as an element of human well-being and security. People living on the U.S. side of the border, many of whom are recent immigrants, need to be empowered to take on more of the responsibility for their own health, whether it is preventive behavior, knowing when and how to seek care, or managing health conditions at the household level, including access to potable water. Improving access to potable water in border communities will lead to improving human security and building resilience and sustainability, a goal worthy of our most diligent effort. 
